# On the Preparation of an Improved Wine of Iron

**Published:** 1864-11

**Authors:** H. N. Draper, J. Whitla


On the Preparation of an Improved Wine of Iron, by H. N. Draper, F. C. S., and Mr. J. Whitla.—The authors first described their observations of the action of light in promoting decomposition of the officinal wine of iron. To prevent this decomposition, which occurs even in the dark, they suggested that ammonia-citrate of iron should replace potassio-tartrate, and that citrate of ammonia should also be added to prevent any slight precipitation that might otherwise occur when the wine was exposed to strong sunlight. The formula proposed was as follows:

| | |
|---|---|
| Ammonia-citrate of Iron, - - - | 160 grains. |
| Crystaline Citrate of Ammonia, - - | 60 " |
| Sherry, - - - - - - - | 1 pint. |

The wine thus prepared was perfectly transparent, and had no disagreeable taste.—[*London Pharm. Journ.* for Oct.—*Journ. of Pharmacy.*

